# Data-driven integration of genome-scale regulatory and metabolic network models

**DOI:** 10.3389/fmicb.2015.00409

**Published:** 2015-05-05

**Authors:** Saheed Imam, Sascha Schäuble, Aaron N. Brooks, Nitin S. Baliga, Nathan D. Price

**Affiliations:** ^1^Institute for Systems BiologySeattle, WA, USA; ^2^Jena University Language and Information Engineering Lab, Friedrich-Schiller-University JenaJena, Germany; ^3^Departments of Biology and Microbiology, University of WashingtonSeattle, WA, USA; ^4^Molecular and Cellular Biology Program, University of WashingtonSeattle, WA, USA; ^5^Lawrence Berkeley National LabBerkeley, CA, USA

**Keywords:** metabolic networks, transcriptional networks, constraint-based modeling, network integration, flux balance analysis, signaling, regulation, metabolism

## Abstract

Microbes are diverse and extremely versatile organisms that play vital roles in all ecological niches. Understanding and harnessing microbial systems will be key to the sustainability of our planet. One approach to improving our knowledge of microbial processes is through data-driven and mechanism-informed computational modeling. Individual models of biological networks (such as metabolism, transcription, and signaling) have played pivotal roles in driving microbial research through the years. These networks, however, are highly interconnected and function in concert—a fact that has led to the development of a variety of approaches aimed at simulating the integrated functions of two or more network types. Though the task of integrating these different models is fraught with new challenges, the large amounts of high-throughput data sets being generated, and algorithms being developed, means that the time is at hand for concerted efforts to build integrated regulatory-metabolic networks in a data-driven fashion. In this perspective, we review current approaches for constructing integrated regulatory-metabolic models and outline new strategies for future development of these network models for any microbial system.

## Introduction

**Figure 1:**
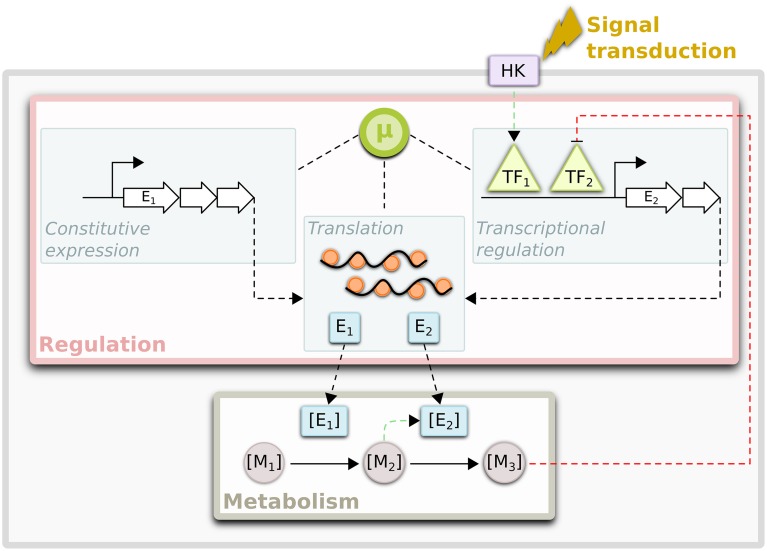
**Interconnections between regulation and metabolism**. Regulation of flux through metabolic networks is achieved by the control of enzyme levels ([E]) and/or activities. Enzyme levels can be controlled transcriptionally via specific regulation of transcription factors (TFs) or via global mechanisms, which depend on factors such as growth rate (μ). The expression levels of constitutively expressed genes may be solely under control of these global mechanisms. In addition, growth rate also has a significant impact on translation rates. The activities of TFs can be modulated by specific metabolites ([M]) or via post-translational modifications by histidine kinases (HK) that sense environmental cues, among other mechanisms. Enzyme activities can also be modulated via post-translational (allosteric) interactions with metabolites. All these networks are dynamic and in constant communication with one another to determine metabolic state of a cell.

Microbial genomes encode a vast repertoire of metabolic pathways that enable physiological adjustment to changing energy sources and nutrient availabilities. The efficient utilization of environmental resources requires selective and timely expression of the metabolic machinery to meet cellular demands. As a consequence, highly interconnected macromolecular networks of metabolic and regulatory components have evolved to control expression of the genome in response to internal and external cues (Figure 1) (Gerosa and Sauer, 2011; Metallo and Vander Heiden, 2013; Chubukov et al., 2014). A primary goal of modern systems biology is to build increasingly accurate representations of these networks that can be used to predict how the macromolecular composition of an organism may change in response to genetic or environmental perturbations. Such models serve as platforms for hypothesis generation that ultimately enable many perturbations to be screened *in silico* before being tested *in vivo*, dramatically accelerating the pace and efficiency of scientific discovery (Tomita, 2001; Bonneau et al., 2007; Oberhardt et al., 2009).

A number of algorithms to construct metabolic, signaling and gene regulatory network models have been developed recently (Thiele et al., 2009; Hyduke and Palsson, 2010; Marbach et al., 2010; Novichkov et al., 2010; Thiele and Palsson, 2010; Yachie et al., 2011). Many were spurred by specific technological advances that enabled comprehensive measurement of microbial cellular components (Thiele et al., 2009; Henry et al., 2010; Thiele and Palsson, 2010; Marbach et al., 2012). These methods have not only been instrumental for contextualizing genome-wide measurements, but have also provided a systems-level perspective of biological organization and function (Oberhardt et al., 2009; Bordbar et al., 2014). Integrated network models that are able to capture these different layers of biological function, on a genome-scale, represent major accomplishments with the potential to revolutionize scientific research (Tomita, 2001).

However, integrating these network models brings about new challenges, both computational and experimental. For instance, algorithms need to be developed to handle the diversity of data types and the various formalisms used to model different biological processes (Machado et al., 2011). Additional challenges also arise from the fact that these processes may occur across vastly different timescales, ranging from milliseconds to weeks. From an experimental standpoint, further technological advancements will be needed to obtain the fine-grained measurements that will be required to build, validate, and refine these models. In this perspective, we briefly review state-of-the-art methods for constructing integrated regulatory-metabolic models, then outline new strategies for constructing data-driven integrated models and suggest how these integrated models could be used to advance basic research, as well as biotechnology.

## Advances in the integration of metabolic and regulatory network models

Kinetic and constraint-based modeling approaches have enabled quantitative modeling of metabolic processes and played key roles in guiding scientific research (Varma and Palsson, 1994; Palsson, 2000; Steuer et al., 2006; Tran et al., 2008; Bakker et al., 2010; Tan and Liao, 2012; Zielinski and Palsson, 2012). Constraint-based metabolic models (CBMs) have proven to be particularly useful as they enable genome-scale modeling of metabolism. However, these purely metabolic models are limited in their ability to capture condition-dependent changes in metabolic activity (Reed, 2012; Machado and Herrgard, 2014). Thus, to incorporate aspects of the regulatory mechanisms that control metabolism, models that integrate CBMs with known or inferred transcriptional regulatory networks (TRNs) have been developed.

To date, only a handful of methods for the genome-scale integration of transcription and metabolism have been described, including regulatory flux balance analysis (rFBA) (Covert and Palsson, 2002), steady-state rFBA (SR-FBA) (Shlomi et al., 2007) and probabilistic regulation of metabolism (PROM) (Chandrasekaran and Price, 2010). The earlier approaches (rFBA and SR-FBA) used Boolean rules to approximate transcriptional control of the metabolic network, permitting only two activity states (on/off) for network components (Covert and Palsson, 2003; Shlomi et al., 2007). With PROM, the Boolean logic is relaxed by introducing probabilistic weights on regulatory influences using gene expression data to estimate the probability that particular TF-gene interactions are functional, allowing for a full range of potential responses from the strength of either activating or repressing regulation (Chandrasekaran and Price, 2010).

These integrated models, however, only consider a static, composite view of a TRN that has dynamic and condition-specific states, thus limiting their utility. To overcome some of these shortcomings, approaches have been developed to identify relevant TRN constraints that allow accurate growth phenotype predictions of a CBM under a given condition, in essence generating condition-specific TRNs (Barua et al., 2010; Chandrasekaran and Price, 2013). Another limitation of these integrated models is that the regulation of metabolic processes occurs at several levels (i.e., transcriptional, post-transcriptional, translational and post-translational), which are not explicitly accounted for in any of these formalisms. As a result, recent efforts have been geared toward integrating some or all of these components into unified models for well-studied microbes (Karr et al., 2012; Lerman et al., 2012; Carrera et al., 2014).

Metabolism and macromolecule expression (ME) models, which integrate stoichiometric representations of gene expression (transcriptional and translational) networks with CBMs, capture important aspects of the mechanisms of macromolecule synthesis (Lerman et al., 2012; O'brien et al., 2013). These models, which impose global growth-related regulatory constraints on metabolism, have been shown to be better predictors of cell phenotypes such as growth, metabolic fluxes and to some extent gene expression levels, than standalone CBMs (O'brien et al., 2013). ME models thus represent a significant advance over CBMs for the holistic modeling of microbial growth. However, ME models currently do not explicitly account for the specific regulatory mechanisms of the TRN or environmental cues, representing an important frontier for enhancing their scope. Recently, Carrera et al. constructed an integrated model for *Escherichia coli* that combines information from its known transcriptional regulatory, signal transduction and metabolic networks, with high-throughput transcriptomics and phenomics data (Carrera et al., 2014). This integrated network was shown to have greater capabilities than CBMs or ME models for prediction of condition-dependent phenotypes, and provides a useful framework for data-driven integration of genome-scale networks.

A major goal of systems biology is the construction of predictive models of the entire cell or organism (Tomita, 2001). One of the first efforts directed toward achieving this was the E-cell platform for simulation of biological processes based on predefined lists of biomolecules, reaction rules and cell environments (Tomita et al., 1999). A significant advance on this front was the construction of the whole cell model of *Mycoplasma genitalium* (Karr et al., 2012). While this model also relies on a very large number of detailed molecular measurements, which are unavailable for most organisms, it provides the first glimpse into the future of full-featured, large-scale integrated models that enable dynamic simulation of cellular processes.

## Toward full-featured integrated models

**Figure 2:**
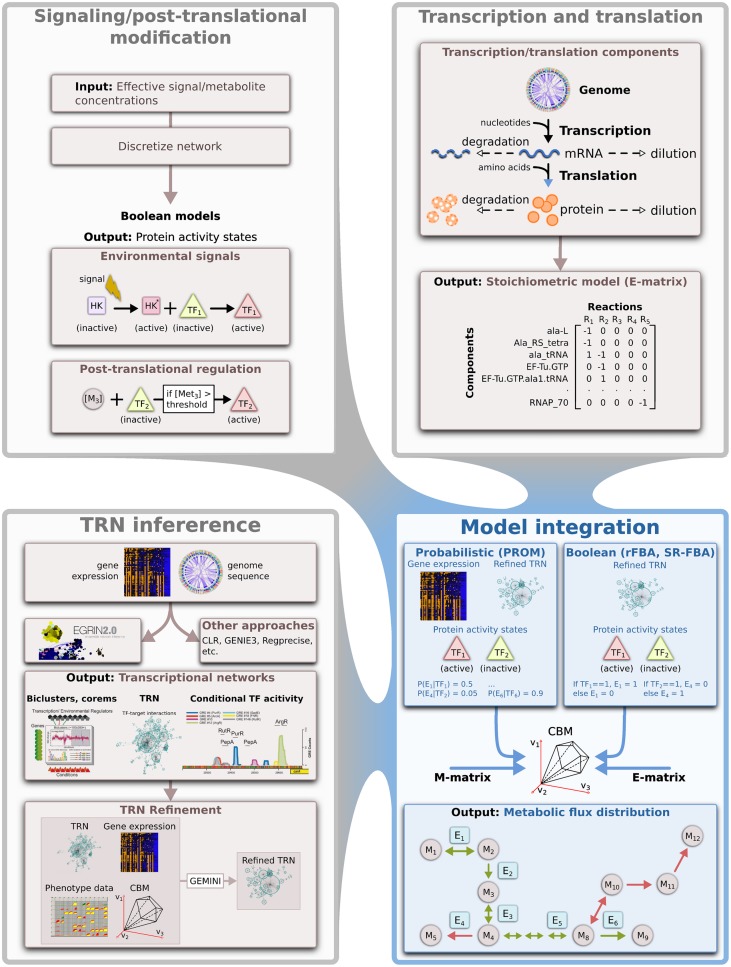
**Modeling and integrating of different biological networks**. An overview of the approaches used to model disparate biological processes and the computational techniques that could be used for integrating some of these network models. HK, histidine kinase; M, metabolite; E, enzyme; TF, transcription factor; TRN, transcriptional regulatory network.

Here we outline the main components that are needed to construct integrated models that capture the key aspects of regulation and metabolism in microbes (Figure 2), with a focus on data-driven approaches that are extensible to any sequenced microbe.

### Genome-scale metabolic models

CBMs enable genome-scale modeling of metabolic networks in the absence of kinetic parameters, and provide a platform for integrating multi-omic datasets. While calculations from CBMs often result in a large solution space, which can include many biologically implausible solutions (Reed and Palsson, 2004; Schellenberger et al., 2011), the addition of biologically relevant constraints can significantly improve their predictive accuracy (Reed, 2012). Manually curated genome-scale CBMs have been constructed for many organisms (Oberhardt et al., 2009; Kim et al., 2012) (Table S1). As many metabolic pathways are broadly conserved, these curated CBMs can serve as scaffolds for automated reconstruction of high-quality organism-specific CBMs for closely related organism using genomic information, conceivably making high-quality CBMs available for any sequenced organism, while minimizing the need for manual curation (Henry et al., 2010; Karp et al., 2010; Swainston et al., 2011; Agren et al., 2013; Benedict et al., 2014). Furthermore, computational tools and databases that facilitate automated identification of biomass components (Tervo and Reed, 2013) and minimal media composition (Richards et al., 2014) should further streamline this process.

### Transcription and translation network models

Growth rate-dependent global regulation functions in concert with TF-controlled specific-regulation to determine the genome-wide expression profiles under a given condition (Berthoumieux et al., 2013) (Figure 1). Global regulation has even been proposed to be the dominant form of regulation under laboratory conditions in some organisms (Price et al., 2013). Because the components of the transcription and translation machinery are relatively well-conserved across bacteria and can be discerned from genomic information, the *E. coli* gene expression network model (Thiele et al., 2009) provides a template for the construction of similar network models for other sequenced bacteria. Thus, we expect that approaches used for accelerating the generation of genome-scale models will eventually be extended to the more complex task of constructing gene-expression networks for other microbes.

### Transcriptional regulatory network (TRN) models

Microbes control the activities and abundance of molecular components to respond quickly to environmental change. A primary mechanism through which microbes exert control over specific cellular processes is through the coordinated transcriptional regulation of gene expression (Gerosa and Sauer, 2011) (Figure 1). Unfortunately, unlike metabolic networks, TRNs are not highly conserved across lineages. Thus, transcriptional regulatory interactions learned in one species may not necessarily exist in others, unless they are related over short phylogenetic distances or share similar lifestyles (Lozada-Chavez et al., 2006; Madan Babu et al., 2006). However, data from high-throughput measurement of global gene expression levels, along with information encoded in the genome of a target organism, can be used for data-driven reconstruction of TRNs (Figure 2) and this has spurred the development of a wide variety of approaches.

The approaches for reconstructing TRN topology (i.e., the set of interactions between TFs and their target genes) vary, ranging from aggregation of experimentally verified interactions (Gama-Castro et al., 2011) to detection of evolutionary conservation among gene targets of related TFs (Novichkov et al., 2010) to data-driven approaches that reverse-engineer TRN topology from relative changes in gene expression (Bonneau et al., 2007; Faith et al., 2007; Huynh-Thu et al., 2010) (Table S1). The advantages and limitations of some of these data-driven approaches have previously been reviewed (De Smet and Marchal, 2010; Marbach et al., 2010). Many of these approaches have also been subjected to unbiased assessments (Stolovitzky et al., 2007; Marbach et al., 2012), systematically identifying their strengths and weaknesses.

To understand TRN function, however, it is also important to know when specific TF-target gene interactions occur. In other words, condition- and/or context-specific interactions determine the consequences of regulation. Such knowledge is particularly important for integrating TRN models with other genome-scale models. Few approaches currently model the condition-specific activities of TFs and their effect on TRNs. A recently published second generation Environmental and Gene Regulatory Influence Network model (EGRIN 2.0) was developed to address this limitation by quantifying the condition-specific regulatory influence of TFs on their target genes and their role in re-organizing the modularity of TRNs for two microbes (Brooks et al., 2014). Since these models specify environmental dependence in addition to topology, TRN models like EGRIN 2.0 are promising candidates for integration with metabolic and other network models.

### Signaling network models

Microbes respond to constantly changing environments by altering their gene expression patterns. Bacteria achieve this coordination through the use of one-component, two-component and extra-cytoplasmic function sigma factor signal transduction systems, which sense stimuli and orchestrate appropriate cellular responses (Ulrich and Zhulin, 2010). While environmental signals that elicit certain transcriptional responses (e.g., catabolite repression, oxidative stress response etc.) have been well-studied (Farr and Kogoma, 1991; Gorke and Stulke, 2008; Chiang and Schellhorn, 2012), many other signaling systems remain uncharacterized.

Even though signaling networks in bacteria are generally simpler than those employed in eukaryotes, reconstruction of intracellular signaling networks still poses a major challenge. As a result, large-scale signaling networks exist for only a few organisms (Covert et al., 2008; Carrera et al., 2014). Since independent discovery and characterization of these signaling systems would be costly and time consuming, it is desirable to predict the effects of environmental changes based on high-throughput datasets. EGRIN provides one approach to link signaling to internal cellular processes (Bonneau et al., 2007). It achieves this by abstract representation of the biological effect of signaling networks as “environmental factors.” These environmental factors can be associated statistically to internal molecular processes, such as transcription. This feature, however, requires meticulous experimental documentation, including direct measurement of the relevant environmental factors (or their proxies). Unfortunately, publicly available datasets are generally poorly annotated and typically not quantitative, limiting the current utility of this approach. Consequently, a greater emphasis should be placed on thorough experimental annotation to facilitate these data-driven approaches.

### Post-translational regulation

Post-translational mechanisms also play a critical role in regulating metabolic flux. For instance, internal ligand concentrations can alter the activities of TFs that regulate associated pathways (Lim et al., 1987; Ramseier et al., 1995; Leyn et al., 2011). Furthermore, the activities of numerous enzymes are controlled via allosteric interactions (Figure 2). Thus, knowledge of these regulatory metabolites, their effective concentrations and their target proteins will be crucial for achieving predictive control.

For model organisms like *E. coli*, a number of these regulatory metabolites and their targets are known and approaches exist for incorporating these into integrated models using Boolean rules and/or ordinary differential equations (Covert and Palsson, 2002; Covert et al., 2008). While some allosteric interactions are widely conserved such as fructose-1,6-bisphosphate (FBP) activation of pyruvate kinase, which is conserved from *E. coli* to humans (Waygood et al., 1976; Jurica et al., 1998; Chubukov et al., 2014), different groups of organisms likely use different strategies and regulatory metabolites. For example, the regulators of glycolysis in γ-Proteobacteria (Cra), α-Proteobacteria (CceR), and β-Proteobacteria (HexR) are post-translationally regulated by FBP, 6-phosphogluconate and 2-keto-3-deoxy-6-phosphogluconate, respectively (Ramseier et al., 1993; Leyn et al., 2011; Imam et al., 2015). Thus, approaches for high-throughput screening of allosteric effectors (Tagore et al., 2008; Li et al., 2010) need to be leveraged to complement standard *in vitro* approaches to identify post-translational interactions. This process could be facilitated by the development of algorithms that borrow from the field of molecular modeling (Lengauer and Rarey, 1996) to assess the potential of protein-ligand interactions across the network.

## Integrating disparate network models

While individual network models have played important roles in improving our understanding of biological systems, recent attention has turned toward integrating them. Such integrated models would encapsulate how regulatory mechanisms control metabolism and how metabolism, in turn, provides feedback regulation on a genome-scale (Figure 1). The motivation for network model integration reflects an acknowledgement that individual models are insufficient to comprehensively describe their respective cellular processes.

One approach to constraining the solution space of CBM predictions is the integration of growth-related constraints on gene expression (i.e., the rates of gene transcription and mRNA translation). Translation and transcriptional network models, which have been constructed for *E. coli* (Thiele et al., 2009; O'brien et al., 2013) and *Thermotoga maritima* (Lerman et al., 2012), including their mathematical formulation and integration with CBMs to generate ME-models, provide a basis for construction of similar ME-models for other microbes based mostly on genomic information. As very few parameters need to be specified for integration of these stoichiometric models, construction of ME-models for any sequenced bacterium should become a relatively straightforward task.

ME-models, however, do not currently account for specific regulatory interactions at gene promoters, which are also known to be important drivers of cellular phenotypes. To build comprehensive models, ME-models need to include regulatory constraints from condition-specific TF-gene interactions (O'brien et al., 2013). However, unlike global transcriptional processes, these may not have straightforward stoichiometric representations. Hence, alternative formulations need to be considered. One possibility could involve leveraging a probabilistic formalism such as PROM for integrating inferred TRN models with ME-models. If such TRN models were developed using EGRIN or related approaches, environmental variables could also be integrated using PROM. Extension of ME-models with TRN information represents an exciting frontier that would provide a platform for simulating metabolism with unprecedented detail.

Integration of signaling information poses some unique challenges. For instance, signaling mechanisms are typically dependent on specific (and often unknown) concentrations of relevant molecules, while constraint-based approaches such as FBA do not deal directly with metabolite concentrations. Furthermore, to generate dynamic quantitative signaling network models, kinetic parameters are required, but these are rarely available. This limits the approaches via which these models can be integrated within the paradigm described above. Thus, qualitative representations of signaling networks using Boolean (Klamt et al., 2006) or stoichiometric (Papin and Palsson, 2004) formalisms need to be adopted for integration of these networks with large-scale regulatory-metabolic models (Figure 2, Table S1). These approaches have the advantage of not requiring specification of kinetic parameters or exact molecule concentrations (which can discretized, Klamt et al., 2006), while still being able to capture fundamental properties of signaling networks.

Other challenges to building integrated models are outlined in Box 1, while approaches that may be useful for validation of such models are discussed in Box 2.

Box 1 **Challenges to constructing integrated regulatory-metabolic models.**

Here we identify some major challenges to building data-driven integrated models of metabolism and regulation. Some of these challenges also represent significant opportunities for algorithmic or experimental breakthroughs.

*Comprehensive discovery and characterization of biological components*. Reconstruction of biological networks requires an exhaustive list of the components and their functions. However, a large fraction (up to 50%) of the predicted proteins across microbial genomes still have unknown functions (Hanson et al., 2009). This missing information can significantly impact the predictive accuracy of systems biology models. While this process of parts identification is significantly facilitated by comparative genomics and related approaches, this can still be a mitigating factor for groups of bacteria that are not yet well studied.

*Greater accuracy of data-driven TRN inference*. While TRN inference has played a crucial role in identification of new TFs and novel regulatory interactions, the predictive accuracy and coverage of TRNs constructed from gene expression data is still relatively low. Even for a well-studied bacterium like *E. coli* for which large compendia of gene expression data exist, state of the art inference approaches only identify a small fraction of the verified interactions in regulonDB with relatively low precision (Marbach et al., 2010, 2012; Gama-Castro et al., 2011). While we anticipate that integration of comparative genomics, constraint-based modeling and other complementary approaches will improve the accuracy and coverage of inferred networks, large gains in predictive accuracy will likely require alternative complementary high-throughput datasets such as ChIP-seq data with tagged TFs (Aldridge et al., 2013; Gasper et al., 2014), DNase I hypersensitivity or genome-wide promoter activity assays.

*High-throughput approaches for identifying signaling events*. As mentioned above, there is a dearth of both experimental and computational approaches for quick screening and identification of potential signaling interactions. Development of approaches in this area would significantly facilitate reconstruction and integration of signaling network models.

*Functional characterization of post-translational modifications*. A vast array of metabolic and regulatory proteins are regulated via post-translational modifications. While post-translational modifications are more prevalent in eukaryotes than bacteria, a large and growing number of these modifications are being identified in bacteria, including phosphorylation (Pietack et al., 2010; Schmidl et al., 2010), succinylation (Zhang et al., 2010) and acetylation (Wang et al., 2010)—and each of these can have major impacts on metabolism. While these modifications can easily be identified by mass spectrometry techniques, determination of their functions, if any, is more challenging. However, by combining metabolic flux analysis with mass spectrometry data collected across varying conditions, insights into the function of some these modification can be determined (Wang et al., 2010), though the cost of such analysis may be prohibitive. Integrating such information using stoichiometric representations would relatively straightforward.

Other challenges such as limitations in availability of quantitative data across conditions, tools for visualization of integrated networks and difficulties in integrating different network formalisms at genome-scale are also important considerations.

Box 2 **Model validation**

Model validation is important both to assess model accuracy and identify shortcomings that can be improved in subsequent versions. However, it is not obvious what validation approaches would be optimal for large-scale integrated models. Traditionally, predictions from CBMs have been validated using substrate utilization and/or gene essentiality data, which has served as a successful approach both for model validation and refinement (Bochner et al., 2001; Feist et al., 2007; Oh et al., 2007; Thiele and Palsson, 2010; Imam et al., 2013). Similarly, initial attempts to validate regulatory-metabolic models have focused on the use of gene essentiality data (Covert et al., 2004; Chandrasekaran and Price, 2010). TRN models, by contrast, have usually been validated by comparison to experimentally derived networks (Stolovitzky et al., 2007; Marbach et al., 2012).

We argue that both of these binary approaches to validation are insufficient to generate key insights that will drive model improvement. Instead, we suggest that quantitative phenotypes may be more appropriate. For instance, deletion of regulatory components such as TFs are typically non-lethal. However, this does not imply that cellular phenotypes are unaffected in these strains. TF deletions may alter growth rates or modify other quantitative cellular phenotypes. In addition, TF deletions may only show their impact across a narrow range of conditions. Thus, simple gene essentiality may be inadequate to assess model performance effectively. More informative would be data from high-throughput growth or fitness assays using deletion mutant libraries (Nichols et al., 2011; Vandersluis et al., 2014) or high-throughput mutagenesis experiments across conditions (Van Opijnen et al., 2009; Khatiwara et al., 2012), which would permit identification and statistical evaluation of genotype-phenotype relationships. Such large-scale datasets should permit robust assessment of the various components of regulatory models and possibly guide the process of model refinement.

## Using integrated models to drive scientific discovery

Construction and analysis of individual large-scale systems biology models has led to important new biological insights about novel pathways, regulatory interactions and mechanistic details (Bonneau et al., 2007; Oberhardt et al., 2009; Hyduke and Palsson, 2010). Given that these networks are highly interconnected, one might expect that analysis of the properties of integrated models will provide new insights into biological phenomena not achievable with individual network models. Such insights could include how novel inferred transcription-regulatory interactions might redirect flux through apparent suboptimal routes in a metabolic network; identification of synthetic rescues/lethal phenotypes in regulatory components; identification of new knowledge gaps that could guide experimental design; or identification of functional roles for previously redundant network components such as dead-end metabolites (Covert et al., 2008). In addition to this, we expect full-featured regulatory-metabolic models will be crucial in driving scientific research in areas such as:

### Metabolic engineering

CBMs have proved to be very useful tools for guiding the design of genetically modified microbial strains with desired characteristics (Alper et al., 2005; Park et al., 2007; Milne et al., 2009). Many approaches have been developed to identify metabolic or genetic interventions that result in these traits (Segre et al., 2002; Burgard et al., 2003; Pharkya et al., 2004; Shlomi et al., 2005; Kim and Reed, 2010). Currently, these approaches do not consider the contribution of regulation on predicted genetic strategies or the benefits of genetic intervention at the regulatory level. Integrated regulatory-metabolic models will provide these capabilities, permitting: (i) rational strain engineering via modification of regulatory components (e.g., over-expression of TFs); (ii) exclusion of metabolic interventions that are inconsistent with the integrated network structure; or (iii) identification of environmental conditions that might facilitate production of desired products. Thus, integrated regulatory-metabolic models could open up several new avenues for modification of cell phenotypes not currently achievable with CBMs.

### Improved network inference

While TRNs inferred from high-throughput data have led to the identification of novel interactions and mechanisms, these approaches are error prone (De Smet and Marchal, 2010; Marbach et al., 2010). Recent analysis has shown that known or inferred TRN topology can be refined to achieve consistency with known phenotypes of a target organisms by integration with CBMs (Chandrasekaran and Price, 2013). For instance, the algorithm GEMINI uses PROM formalism to integrate TRN models with CBMs, and then attempts to identify global regulatory interactions that are consistent with condition-specific growth phenotypes, thereby refining the TRN and potentially improving its quality (Chandrasekaran and Price, 2013). While GEMINI was originally used as a post-processing step, there exists the potential of incorporating this or similar approaches into the TRN inference workflow itself to provide inline network refinement (Figure 2).

While a few applications of integrated models have been listed here, this is far from exhaustive and the applications will evolve as new data types and algorithms are developed.

## Concluding remarks

One of the aims of systems biology is to convert system-wide measurements into systems-level biological insight. Computational models that capture the core aspects of biological complexity will be pivotal to achieving this goal. Models of metabolism and regulation can be built from a combination of genomic information, high-throughput measurements, and prior knowledge for any cultured organism. Integrating these models will provide deeper insight into fundamental cellular processes and help contextualize high-throughput experiments.

While full-featured integrated models will be useful to generate biological hypotheses, guide experimental designs and drive biotechnology applications, the level of detail at which these processes are represented within the model will depend on the proposed application. Although additional layers of biological complexity could be included *ad infinitum* to make a model more closely resemble the reality, greater complexity does not necessarily translate into greater utility.

### Conflict of interest statement

The authors declare that the research was conducted in the absence of any commercial or financial relationships that could be construed as a potential conflict of interest.

## Abbreviations

FBA: Flux balance analysis
rFBA: Regulatory flux balance analysis
SR-FBA: Steady-state regulatory flux balance analysis
PROM: Probabilistic regulation of metabolism
CBM: Constraint-based metabolic model
TRN: Transcriptional regulatory network
ME: Metabolism and macromolecule expression
EGRIN: Environmental and Gene Regulatory Influence Network model
FBP: Fructose-1,6-bisphosphate
GEMINI: Gene Expression and Metabolism Integrated for Network Inference
ChIP-seq: Chromatin immunoprecipitation followed by high-throughput sequencing.
